# ^6^Li-loaded directionally sensitive anti-neutrino detector for possible geo-neutrinographic imaging applications

**DOI:** 10.1038/srep04708

**Published:** 2014-04-24

**Authors:** H. K. M. Tanaka, H. Watanabe

**Affiliations:** 1 Earthquake Research Institute, The University of Tokyo, 1-1-1 Yayoi, Bunkyo, 113-0032 Tokyo, Japan; 2 Research Center for Neutrino Science, Tohoku University, 6-3 Aoba, Sendai, 980-8578 Miyagi, Japan

## Abstract

Despite the latent and unique benefits of imaging uranium and thorium's distribution in the earth's interior, previously proposed experimental techniques used to identify the incoming geo-neutrino's direction are not applicable to practical imaging due to the high miss-identification in a neutrino's track reconstruction. After performing experimental studies and Monte-Carlo simulations, we confirmed that a significant improvement is possible in neutrino tracking identification with a ^6^Li-loaded neutrino detector. For possible imaging applications, we also explore the feasibility of producing geo-neutrinographic images of gigantic magmatic reservoirs and deep structure in the mantle. We anticipate and plan to apply these newly designed detectors to radiographic imaging of the Earth's interior, monitoring of nuclear reactors, and tracking astrophysical sources of neutrinos.

Geo-neutrino observations have recently been developing to the extent that we have the capacity to collect data regarding the elemental structure of the deep earth^1^,^2^. Whereas the chemical composition of the outermost earth's crust can be sampled directly, geo-neutrino observations reveal the amount of heavy elements, uranium (U) and thorium (Th), deep inside the earth and can be used to determine its composition by taking advantage of the fact that the geo-neutrino flux can be calculated directly from knowing the mass (*M*) of U and Th in the Earth: 7.46 × 10^7^ × *M* kg and 1.62 × 10^7^ × *M* kg neutrinos per second for U and Th respectively (without considering neutrino oscillation)^3^ (Fiorentini et al. 2010). Geo-neutrino observations have the potential to map out the distribution of U and Th inside the Earth by tracking the direction of incoming geo-neutrinos. However, we do not have the technology to track the direction of incoming geo-neutrinos at present.

Recent progress in muography (the use of muons as a radiographic probe) has imaged density structures inside volcanoes in Japan^4^, France^5^ and Italy^6^ as well as seismic faults^7^ with higher spatial resolution than possible with the conventional geophysical techniques by measuring the arrival directions and counting the cosmic ray muon events after they pass through the target volume. Recent geo-neutrino observations have already shown that geo-neutrinos may similarly produce results that support and clarify the current concerns of earth science such as: estimating the radiogenic heat of contribution to the surface heat flux^8^; constraining existing earth's compositional estimates^9^; and clarifying the origin of low shear velocity regions found at and rising from the core mantle boundary (CMB)^10^. However, (unlike muography detectors) the current geo-neutrino detectors are still under development because they do not have the sensitivity to detect the arrival direction of neutrinos. The directional sensitivities in anti-neutrino measurements has been theoretically and experimentally studied in the Goesgen^11^ (Boehm, 2000), CHOOZ^12^ and Double CHOOZ^13^ (Caden 2012) experiments so far. These reactor experiments attempted to conduct directional (reactor) neutrino measurements in conjunction with nearby reactor experiments by utilizing the fact that the net momentum transmitted to the neutron and positron by the incoming neutrino statistically biases the positron annihilation and the neutron capture locations^12^. However, their detectors do not have a satisfactory angular resolution for mapping geo-neutrino sources (e.g. in the CHOOZ experiment, ~50% of the tracks are miss-identified as tracks originating from the opposite direction^12^).

A low U and Th concentration in the mantle is related to Earth modeling involving chondritic meteorites, which have an abundance of U and Th of 1 × 10^−2^ and 4 × 10^−2^ ppm, respectively. Mantle uranium content has only 10^−3^–10^−2^ ppm, while crustal rock has 1.4 ppm^14^. Subducted oceanic plates, which are relatively poor U and Th concentrations in comparison to the continental crust, begin melting once reaching a depths of about 80–200 km^15^. At these levels (100–200 km), hydrous partial melting of the ocean crust occurs, resulting in hydrous, alkali-rich silicic magmas that permeate the overlying mantle wedge, fluxing the mantle beneath the volcanic arc. These magmas rise and react with overlying lithospheric mantle and crust, often enriching these melts in incompatible elements like U and Th, as these large ions fit poorly into many crystal lattices. As a result, the concentrations of U and Th in these melt diapirs increase as they rise to the surface as they differentiate toward granitic magma. Overall this process leads to the evolved state of the continental crust with enrichments of Th and U, the heat producing elements, towards the surface.

In this work, we propose a novel method to map out the U and Th distribution inside the Earth utilizing lithium (^6^Li)-loaded directional geo-neutrino detectors. Based on the outcome of our experimental modeling of the detector's angular resolution, it is anticipated that only the ^6^Li-loaded detector would provide radiographic (neutrinographic) images of U and Th distribution. In the following sections, Monte-Carlo simulation results obtained from tracking the neutrino trajectories with a ^6^Li -loaded LS detector will be discussed along with the outcome of our high-resolution LS detector model experiments as well as the simulated image showing the hypothetical U and Th distribution inside a magmatic reservoir that was imaged with seismic tomography by Matsubara et al (2000)^16^.

## Results

### Principle of directional geo-neutrino observation

Beta-decays of radionuclides ^238^U, ^235^U, ^232^Th, and ^40^K and others inside the earth produce low energy anti-electron neutrinos. The generated neutrinos traverse through the earth without being disturbed due to their lack of charge and extremely small interaction cross section with matter. These neutrinos are called geo-neutrinos. The flux of geo-neutrinos (*φ*) observed with a detector located a distance *r* from a radioactive reservoir distributed in a spatial domain *V* can be calculated by:
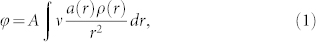
where *a*(*r*) is the concentration of the radionuclides and *ρ*(*r*) is the rock density. *A* is the elementary dependent factor that is expressed as a product of the number of antineutrinos per decay chain, decay constant, abundance of radioactive isotopes, and average survival probability.

The neutrino energies attributed to ^238^U decay chain and to ^232^Th decay chain are all below 3.3 MeV. A relationship between energy of the incoming neutrino and the resolving power has been developed by Vogel and Beacom^17^ in terms of positron and neutron emission angles (with respect to the neutrino direction). However, directional measurements of neutrinos with these energies have never been successful at satisfactory levels because the neutron capture point cannot be well identified although they retain the directional information of the incoming neutrinos. Angular resolution of geo-neutrino tracking depends on how precisely one can determine the vertex points where the inverse beta decay reaction (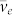 + *p* → *n* + *e*^+^) occurs in a liquid scintillation (LS) detector.

The improvement of directional geo-neutrino observations will be achieved by (A) positioning the e^+^ annihilation point^17^, and (B) shortening the neutron capture range. (A) can be handled by utilizing high position-resolution detectors for precisely measuring the position of the 2 annihilation gamma rays emitted in opposite directions. In the case of (B), if the neutron capture range is shortened, scatterings during the thermalization process are then suppressed, allowing one to measure the initial travel direction of the neutron. A LS detector that incorporates an isotope with a large thermal-neutron capturing cross section enhances its directionality potential by reducing the capture time for the neutron and improves the capability of identifying the initial interaction position. Such detectors at Palo Verde^18^ and CHOOZ^19^ were the first experiments to make directional measurements of anti-electron neutrinos originated from nuclear reactors. Although the CHOOZ detector had a superior directional sensitivity, its tracking miss-identification remains too excessive (73% for >60° and 45% for >90°) to apply to prospective directional geo-neutrino observations^19^. In these experiments, gadolinium (Gd), which produces high-energy capture gamma rays, was doped into the LS, however, these gamma rays are diffuse and will mask the neutron capture point, hence degrading the tracking quality.

However, unlike Gd, lithium (^6^Li) emits a triton instead of gamma rays when the neutron is captured on ^6^Li: ^6^Li + *n* → *t* (2.73 MeV) + *α* (2.05 MeV), and therefore in comparison to other methods, gamma rays do not interfere as much with the detection of the neutron capture point. Its natural abundance and neutron capture cross section are summarized in Table 1. Those for ^10^B (as another candidate dopant) are also shown in this table.

### Model experiments for positioning the annihilation gamma-rays

**Figure 1:**
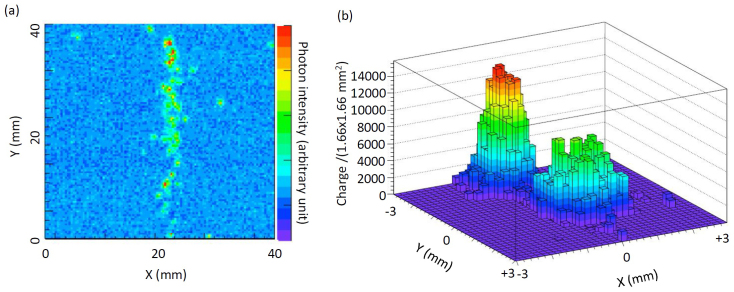
Quality of the imaging detector. (a), imaging of a cosmic ray muon track and (b), the bright spot separation of ^60^Co gamma rays.

The positioning resolution of the gamma rays generated via the prompt positron's annihilation was experimentally modeled. Fig. 1 (a) shows the quality of the imaging detector that we developed for the directional geo-neutrino observations. The image shows the trajectory of a muon in the LS. As can be clearly seen in this figure, the trajectory is determined with the precision of <±2.5 cm. In order to confirm the positioning resolution of gamma rays more quantitatively, we created a gamma-ray beam by collimating the gamma rays from the ^60^Co radiation checking source with a lead (Pb) plate. In order to visually demonstrate the quality of the positioning, a pin hole (with a diameter of 10 mm) was created in the Pb plate, and a bright spot was measured. Fig. 1 (b) shows two bright spots measured at different positions of the Pb plate (the center to center distance is 20 mm). The best fitting gives that the positioning resolution is 8.7 mm (at a 1σ CL). Our result confirms that the present positioning resolution of the geo-neutrino detector can be improved to at least 2 cm/(*E* MeV)^0.5^ for the small-size detector (with a volume less than a few m^3^).

### Modelling of the angular resolution

**Figure 2:**
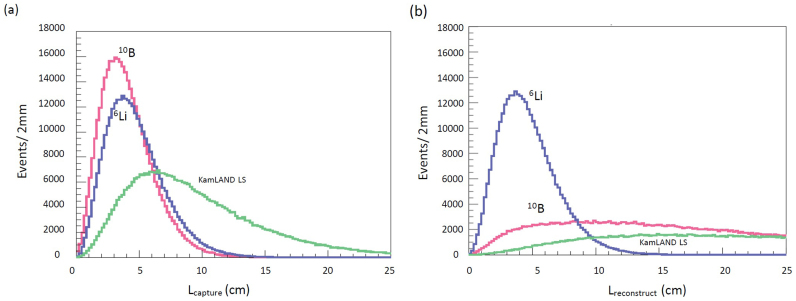
Monte-Carlo simulation results. (a), the distribution of the neutron capture positions (*L*_capture_), and (b), reconstructed neutron capture positions (*L*_reconstruct_). The plots show the results for ^10^B (red) and ^6^Li (blue) loaded liquid scintillator (LS) and KamLAND LS (green) as a function of the distance from the point where the inverse beta decay reaction occurred.

Fig. 2 compares the Monte-Carlo simulated neutron capture (a) and reconstructed points (b) for ^10^B and ^6^Li loaded and Kamland LS. The neutron capture point refers to the distance (*L*_capture_) of which the neutron traverses right after the inverse beta decay reaction (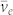 + *P* → *e*^+^ + *n*) until being captured by the given elements; the reconstructed point refers to the reconstructed distance (*L*_reconstruct_) between the reacted proton and the point where the charged particle is emitted. The charged particles include alpha particle, triton nucleus, and gamma-ray recoil particles. Since the neutron capture cross section is largest for ^10^B, the thermal diffusion effect is minimized. The typical distance for neutron capture is 3.0 cm, 4.4 cm, and 6.5 cm for ^10^B (1.0wt%), ^6^Li (0.15wt%) loaded, and KamLAND LS respectively. As can be seen in the figure, only the ^6^Li loaded LS is capable of retaining the neutron capture position information while the neutron capture on ^10^B also emits alpha particle, but the signal is masked by the subsequently emitted gamma-rays. The typical value for the reconstructed distance is 12.8 cm, 4.4 cm, and 15.0 cm for ^10^B, ^6^Li loaded, and KamLAND LS respectively.

**Figure 3:**
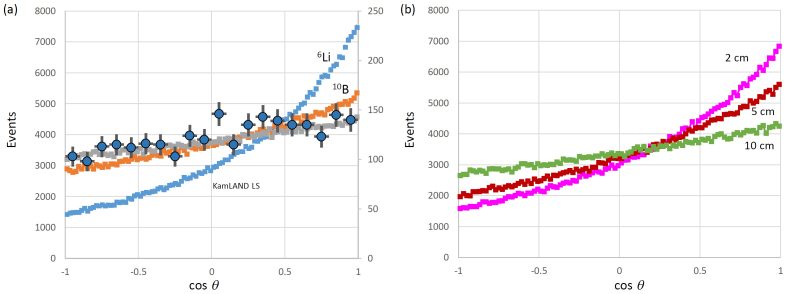
Comparisons of the Monte-Carlo simulation results. The angular resolutions are calculated for (a), ^10^B, ^6^Li loaded, KamLAND liquid scintillator, and (b), ^6^Li loaded (0.15wt%) liquid scintillator with different positioning resolutions of the imaging detector (2 cm (MeV)^−2^, 5 cm (MeV)^−2^, and 10 cm (MeV)^−2^). The data from CHOOZ reactor experiments are also plotted in (a). Left and right axes are “events” (as units) and the right axis is only for CHOOZ data, whereas left is for the other three plotted datasets. The error bars indicate the 1σ standard deviation.

By connecting the neutrino-reacted point and the reconstructed point, the angular resolution of incoming geo-neutrinos was estimated. Fig. 3 (a) compares the angular resolution calculated for ^10^B (1.0wt%), ^6^Li (0.15wt%) loaded, and KamLAND LS. The neutrino injection direction is *θ* = 180° (and *θ* = -180° refers to the opposite direction to the neutrino injection). The plot also compares the actual data as obtained with the CHOOZ reactor experiment^19^. If we define the asymmetry as (*A*_+_ − *A*_−_)/(*A*_+_ + *A*_−_), where *A* is the number of events integrated over a positive and negative angle region in Fig.3 (a), and the positive and negative symbol represents positive and negative angle regions, respectively, the estimated asymmetry for ^10^B, ^6^Li loaded, and KamLAND LS are 0.148, 0.391 and 0.079, respectively. The simulated values are summarized in Table 2. Fig. 3(b) shows the angular resolution as a function of the positioning resolutions of positron annihilations. Lower positioning resolutions blur the neutrino-reacted point, and thus the tracking quality is degraded. As can be clearly seen in this figure, the angular resolution strongly depends on the positioning resolution of the detector. In this calculation, any background noises to create fake tracks are not considered.

## Discussion

### Imaging a gigantic magmatic reservoir underneath the Hida Mountains

**Figure 4:**
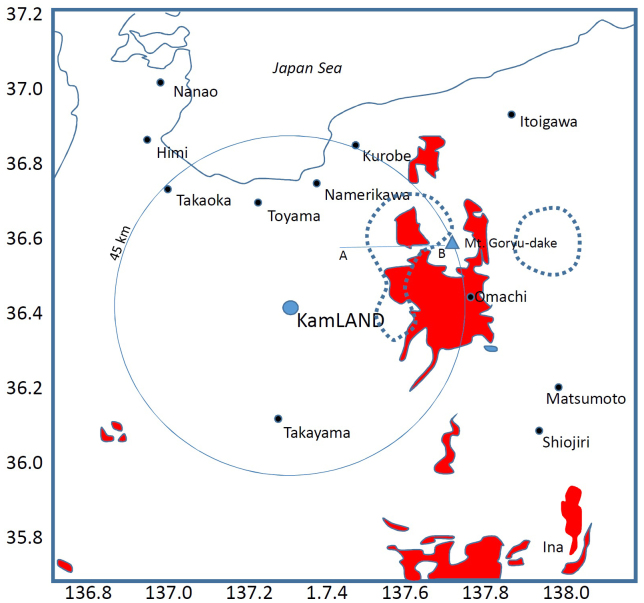
Geochemical map around the hypothetical magma chamber. The map shows the region (red) where the U and Th are highly concentrated (above 5 and 20 ppm)^22^. The dotted lines indicate the Low-*V*_p_ region. Inside the lines, *V*_p_ of 5 km/s was observed. The vertical cross sectional model shown in Fig. 6 is drawn along the A–B line in this map. The circle shows the distance of 45 km from the KamLAND site that intersects Mt. Goryudake. The topographic information along this circle is shown in Fig. 6. H.K.M.T., one of the authors, drew the map and holds the copyright.

**Figure 5:**
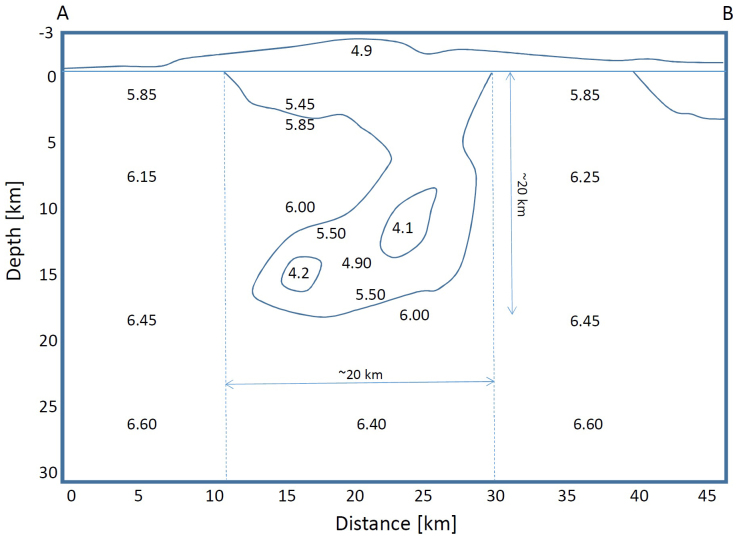
Seismic velocity structure around the hypothetical magma chamber. The *V*_p_ model is based on the seismic tomography conducted by Matsubara et al (2000)^14^. The low velocity zone (<5.5 km/s) was interpreted to be the magma plumbing system, which is located right above the upper/lower crustal interface and underneath the Hida Mountains.

To test the resolving power of our ^6^Li loaded LS detector, we investigated the radiographic appearance of a hypothetical magma chamber. For this, we chose the seismic low-velocity zone found underneath the Hida Mountains, Japan as an example. The Hida Mountains are located in the northern Japan Alps, at the junction of the northeastern and southwestern Japan arc. Katsumata et al. (1995)^20^ first observed extreme attenuation in seismic waves propagated underneath the Hida Mountains, and subsequently Sakai et al. (1996)^21^ concluded that the uppermost low velocity zone is located at a depth shallower than 20 km. A seismic tomographic survey^16^ recognized two discreet areas immediately east of KamLAND that have P-wave velocity profiles lower than 5.5 km/s, with areas outlined in Figure 4. Using a comparison of *V*_p_ with *V*_p_/*V*_s_ ratio the low velocity zone was identified as partially molten rock (Fig. 5), likely a felsic magma^16^. The estimated size of the magma chamber from this study was 16000 km^3^.

Assuming that the average U and Th concentrations of these low velocity regions are comparable to the surface value (5 ppm and 20 ppm) (Fig. 4), it can be estimated that the anticipated contribution of the magma chamber to the neutrino flux (which would pass through the KamLAND site) is 1.5 × 10^5^ (2.0 TNU (events per 10^32^ protons year that is equivalent to ~1 kton LS)) and 1.2 × 10^5^ neutrinos cm^−2^sec^−1^ (1.6 TNU) from the U and Th decays respectively. This value is ~10% of the entire geo-neutrino flux expected to traverse through the KamLAND detector (38.5 ± 7.7 TNU)^22^.

**Figure 6:**
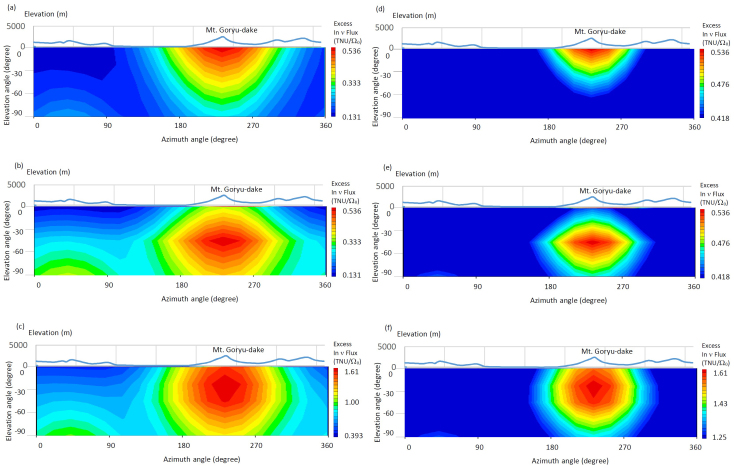
Geo-neutrinographic image of the hypothetical magma chamber. The plots show images for different U and Th concentration models. (a), U and Th are concentrated at 5 ppm and 20 ppm within 1/3 upper part. (b), they are concentrated within 1/3 lower part. (c), they are uniformly distributed over entire region of the reservoir. The images are shown at different lower cutoff values: minimum flux (left panels) and moderate flux (right panels). The distribution is shown as an excess in geo-neutrino flux per given solid angle (Ω_0_ = π/2 sr in this plot). The topographic profile is included.

Based on our modeling of the detector's angular resolution, we produced a geo-neutrinographic image of the magma chamber underneath the Hida Mountains for different U and Th concentration distribution. The detector was assumed to be located at the KamLAND site. Fig. 6 shows the simulated excess in geo-neutrino flux beneath the Hida Mountains with the assumption that the U and Th are concentrated within 1/3 upper part (a), 1/3 lower part (b), and distributed over entire region of the reservoir at the same concentration. The topographic profile along the blue circle shown in Fig. 5 is also plotted for reference. The crustal projection image was produced by taking a moving average of the values within the range of −45° < *θ* < 45° and −45° < *ϕ* <45°, and with an interval of 20° and 10° for the azimuth and elevation angles, respectively and thus, the viewing solid angle (Ω_0_) is π/2 sr (that corresponds to a 1σ angular resolution of our ^6^Li loaded LS detector) for each bin. Assuming the geo-neutrino flux arrives isotropically (except for the neutrinos from our hypothetical magma chamber), the geo-neutrino intensity at the KamLAND site will be ~10 TNU Ω_0_^−1^ whereas an excess in the geo-neutrino flux from the direction of 230 mrad < *ϕ* < 270 mrad measures 2.25 TNU Ω_0_^−1^. This means that the directional geo-neutrino measurements gives us not only the source distribution but also a higher signal to noise ratio (13% → 25%), with the signal being the hypothetical magma chamber, the primary source of these geo-neutrinos and the noise being geo-neutrinos from other sources.

This result reveals that a 3-kton detector records *I*_magma_ ~ 5 Ω_0_^−1^ yr^−1^ at the maximum, where *I*_magma_ is the flux contribution from the hypothetical magma chamber. Since the total geo-neutrino intensity at KamLAND is ~10 TNU Ω_0_^−1^, the detector counts *I*_geo_ ~ 30 Ω_0_^−1^ yr^−1^. *I*_magma_ can be therefore separated from *I*_geo_ at ~3σ confidence level in 10 years. In this simulation, the assumed detector is situated at the KamLAND site (>1 km below the surface) to attenuate near surface background that include muogenic ^8^He and ^9^Li as well as cosmic electromagnetic components and fast neutrons.

The global average U and Th concentrations predicted for the upper crust (2.7 and 10.5)^23^, which is ~2 times less abundant than that observed at the surface of the Hida mountains. The abundances of these and other highly incompatible elements that are enriched in the upper crust follow a log-normal distribution^24^ meaning that there is a reasonable probability that there are other geo-neutrino flux sources of comparable or greater strength to that hosted in the magma chamber. Our technique only resolves integrated amounts of U and Th along neutrino paths, and thus cannot distinguish these sources. There are also electron anti-neutrinos originating from reactors (although the KamLAND location has a much reduced reactor signal since the shutdown of Japanese reactors after the 2011 Tohoku earthquake). To solve these problems, it might be useful to conduct multi-directional radiographic measurements with two or more directionally sensitive neutrino detectors encircling the target. Tanaka *et al*. (2011)^25^ applied this technique to muographic observations in a volcano to reconstruct the three-dimensional structure in Asama volcano, Japan. However, further investigations are necessary to confirm the applicability of this technique to geo-neutrinography.

We demonstrated the resolving power of a ^6^Li-loaded, direction sensitive anti-neutrino detector by imaging a hypothetical magmatic reservoir. A ^6^Li-loaded direction sensitive anti-neutrino detector is an effective solution to the challenges that have been attempted by other groups. Geo-neutrinography offers an independent and novel method for exploring felsic magma chambers. Moreover, the technique is also applicable to resolving crust versus mantle (horizontal vs vertical) flux contributions, as well as neutrinographic imaging of the Earth's interior. Sramek *et al*. (2013)^10^ estimated that the flux contribution from two large, low shear velocity provinces (LLSVPs)^26^ is up to ~2.0 × 10^5^ neutrinos cm^−2^sec^−1^ (at places right above LLSVPs) which is comparable to the flux contribution from the hypothetical magma chamber. Such excess fluxes can be geo-neutrinographically imaged with our directionally sensitive technique working in conjunction with a future ocean based geo-neutrino detector^27^. This new technology also has additional applications in the monitoring of nuclear reactors, and tracking astrophysical sources of neutrinos. Results from our model experiments and simulations show that a ^6^Li loaded LS detector has the potential to be a candidate for the next generation directionally sensitive geo-neutrino detector, although much work remains before it can be fully developed. Our future work includes (A) consideration of near surface background to confirm whether our proposed signature is resolvable with future detectors in ideal and accessible locations and a useful signal-to-noise against backgrounds, and (B) investigation of the possibility of using fast pixel timing (≪100 ps) photon detectors such as Large-Area Picosecond Photo-Detectors (LAPPD) for further noise reduction.

## Methods

### Imaging detector

The conventional geo-neutrino detector does not have a good vertex resolution (e.g., ~130 mm (*E* MeV)^−0.5^ in the case of KamLAND). If a detector's resolution is not sufficient to separate annihilation gamma rays, the tracking quality is significantly degraded. In order to increase the vertex resolution, an image intensifier (II) or multi-anode photomultiplier tube (MAPMT) can be used for an imaging detector. The photons generated along the particle's trajectories generated in the LS are made to converge towards the imaging device via an optical lens.

**Figure 7:**
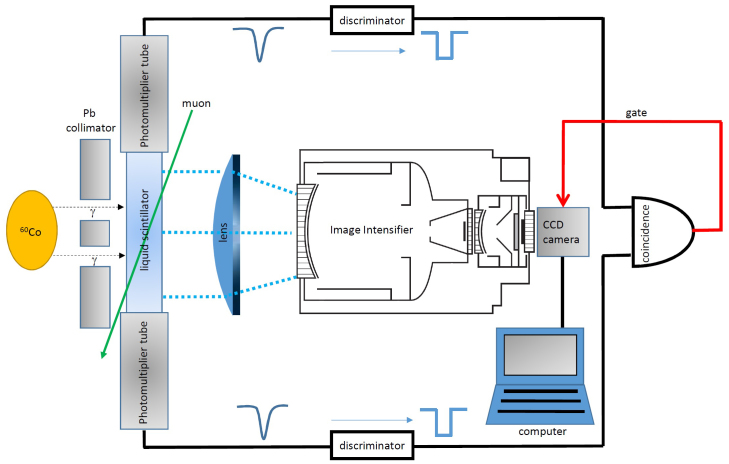
Experimental setup of the present model experiment. Two photomultiplier tubes (PMTs) were used for making a trigger signal for capturing a scintillation image intensified by the image intensifier. Discriminators are used to convert analog signals from PMTs to digital signals.

In order to confirm the quality of the vertex resolution with our II-based imaging detector, we performed a test measurement according to the following steps: (a) a large diameter II (Hamamatsu V5502UX/V1366PGX/CCD) was coupled with a CCD (Charged Coupled Device) camera (Hamamatsu C9300-201); (b) A wavelength shifter (WLS) was loaded into the LS with a dimension of 6 cm^W^ × 6 cm^L^ × 3 cm^D^ so that the wavelength of the scintillation light (λ = 370 nm) matched the maximum acceptance of the II (QE of 12% at λ = 420 nm); and (c) an Achromatic lens (focal length of 60 mm and diameter of 40 mm) was inserted between the LS and II in order to converge the scintillation photons to the II. The CCD is triggered by the signal from two PMTs attached to the side of the LS. The experimental set up is shown in Fig. 7.

### Monte Carlo modeling

Thus far, the ^6^Li loaded liquid scintillator was utilized for fast neutron (with energies above 1 MeV) detection^28^,^29^, and was successfully operated in order to determine the time range necessary for the neutron to be captured by ^6^Li, the results of which were in agreement with their Monte Carlo simulations. Based on these experimental results, we performed Monte Carlo simulations to estimate the angular resolution and the miss-identification rate for tracking geo-neutrinos.

Simulations of the thermalization and capture processes of neutrons were conducted with Geant4^30^. For reactions initiated by neutrons with energies lower than 20 MeV, we used the packages called Geant 4 Neutron Data Library (G4NDL) and Evaluated Nuclear Data File (ENDF). These packages include the processes of elastic and inelastic reactions, neutron capture, and several fission models. The simulation procedure includes (a) filling an infinite volume with liquid scintillator; (b) loading ^6^Li and^10^B with different concentrations, (c) injecting neutrons according to the energy spectrum from the anti-beta decay process, and (d) tracking these neutrons by connecting the points where the positron is annihilated (also including the points of which gamma-ray recoil particles create) and the neutron-initiated alpha (and triton) is emitted. Two 511 keV gamma rays are generated when a positron is annihilated. Calculations were performed for different given positioning resolutions.

### Simulation of geo-neutrinographic imaging of a magma chamber

The geo-neutrino flux is most sensitive to variations in the total uranium mass in the upper continental crust. Moreover, the contribution of the geo-neutrino flux from the volume covered within 50 km from the KamLAND site is 25% of the entire geo-neutrino flux. From Eq. (1), the total neutrino flux from *M* tons of U and Th at a distance of *L* km from the detector is given by integrating following equations over *r* within the volume of the reservoir:
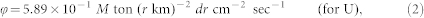

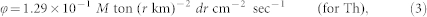
or
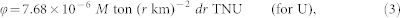

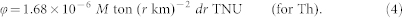


For the hypothetical magma chamber underneath the Hida mountain range in Japan, the following simulation procedure was undertaken: (a) Based on the geochemical map (showing the elemental distribution of the surface) distributed by GSJ (The Geological Survey of Japan)^31^ and the modeled vertical cross section of Matsubara's velocity structure underneath the Hida mountain range^16^, the three dimensional shape of the magmatic reservoir was modeled. The modeled reservoir volume was found to be 16000 km^3^ (800 km^2^ in surface area and 20 km in depth), and the center of the volume is located ~30 km from the KamLAND detector; (b) the average U and Th concentration of the modeled low velocity region was assumed to be the same as the rock sample collected above this anomaly (5 ppm and 20 ppm); (c) by integrating Eqs. (2-1) and (2-2) or (3-1) and (3-2) over *r* within the modeled volume, and (d) by employing the geochemical structural model adopted by Enomoto et al. (2007)^22^ for values in the area excluding the low velocity region, (e) the azimuthal distribution of the geo-neutrino flux is mapped out by applying the results of the Monte-Carlo modeled angular resolution of the detector, which is described in the previous section.

### Statistical uncertainties in the measurements

The entire geo-neutrino flux expected to traverse through the KamLAND site is estimated to be 38.5 ± 7.7 TNU^22^. This flux would correspond to ~40 geo-neutrino events if recorded over the period of a fully efficient, year-long exposure of a 1-kton detector. A 1σ angular resolution of our ^6^Li loaded LS detector is Ω_0_≡ π/2 sr. Since the geo-neutrino flux does not have a strong directional dependence at KamLAND site if we measure it with this angular resolution (which varies by a factor of ~20% between elevation angles of −45 ± 45° and −90 ± 45°), approximately 10 kton^−1^ yr^−1^ Ω_0_^−1^ geo-neutrinos are recorded while simultaneously 1.1 × 10^−4^ km^−3^ kton^−1^ yr^−1^ Ω_0_^−1^ magmagenic neutrinos are recorded by employing a model dependent on U and Th average concentration being 5 and 20 ppm respectively (the unit km^3^ refers to the volume of the magma). Therefore, the amount of magma required to separate magmagenic neutrinos from geo-neutrinos at 1σ, 2σ, and 3σ confidence levels with a 30-kton yr detector are 5.3 × 10^3^, 1.1 × 10^4^, and 1.7 × 10^4^ km^3^, respectively.

## Author Contributions

H.K., M.T. and H.W. wrote the main manuscript text. H.K., M.T prepared figures 4-7. H.W. prepared figures 1-3. All authors reviewed the manuscript.

## Figures and Tables

**Table 1:** Natural abundance and the neutron capture cross section of the candidate elements: boron and lithium^32^. The neutron capture cross section of ^1^H is 0.3 barn

Element	Natural Abundance (%)	Cross section (barn)
B	-	767
^10^B	20	3835
^11^B	80	0.0055
Li	-	70.5
^6^Li	7.59	940
^7^Li	92.41	0.0454

**Table 2:** Monte-Carlo simulated direction sensitivities for different candidate elements: boron and lithium. The result for the KamLAND LS is also shown. The definition of the asymmetry is described in the text

Element	Asymmetry	miss-identification rate (θ>90°)
KamLAND LS	0.079	46.0
^10^B	0.148	42.6
^6^Li	0.391	30.4
Gd	0.097	45.0
